# Earlobe cleft reconstructive surgery

**DOI:** 10.1016/S1808-8694(15)30989-7

**Published:** 2015-10-19

**Authors:** Lucas Gomes Patrocínio, Rodrigo Márcio Morais, José Edmundo Pereira, José Antônio Patrocínio

**Affiliations:** a MD, Otorhinolaryngology Resident - Medical School of the Federal University of Uberlândia; b MD, Otorhinolaryngology Resident - Medical School of the Federal University of Uberlândia; c Dermatologist Surgeon, head of the Dermatology Department - Medical School of the Federal University of Uberlândia; d Full Professor, Head of the Department of Otorhinolaryngology - Medical School of the Federal University of Uberlândia. Department of Otorhinolaryngology - Santa Genoveva Hospital and University Hospital - Federal University of Uberlândia, Uberlândia, Minas Gerais, Brazil

**Keywords:** ear deformities, esthetics, reconstructive surgical procedures

## Abstract

The earlobe occupies a unique position among facial structures and has its own importance when we consider the secular tradition of wearing decorations and jewels on it.

**Aim:**

To present and discuss the technique used in the Departments of Otolaryngology of the Federal University of Uberlândia (FAMED-UFU) and the Hospital Santa Genoveva, in the treatment of earlobe clefts.

**Patient and Methods:**

Twenty-five patients (35 ears) with earlobe clefts were evaluated, from January 2003 to May 2005. In all these cases we used the technique we call “Surgery of the Ear Ring “.

**Results:**

Of the 35 cases, 32 presented satisfactory results, 1 presented with an aesthetic deficit only noticed by the surgeon, and 2 presented aesthetic deficits noticed by both the patient and surgeon, needing a “second look” surgery. In these, there was a notch in the lower free border. The other case with deficit was a functional one caused by the closing of the ear lobe hole.

**Conclusions:**

We consider this technique an innovative one, of easy accomplishment, and with good aesthetic and functional results. Therefore, it is the authors’ preferred technique for the correction of earlobe clefts.

## INTRODUCTION

The ear lobe occupies a unique position among facial structures and is particularly important due to the secular tradition of people wearing decoration and jewellery in this place. This issue of perforating the ear lobe comes from time immemorial and, depending on the culture it happens even as a social obligation. Latin-American cultures have routinely pierced the ears of newborn baby girls, to differentiate them from males. In the tribes of Ivan and Kayan, in Africa, ear lobes were decorated with large and heavy earrings, thus causing an enlargement and elongation of the lobe hole^1^.

Moreover, there is a current trend of using increasingly more decorations in this region, causing a greater local tension and structural alterations arising from these new habits, even in males. All of this has motivated a greater number of patients to seek specialized treatment for cosmetic alterations in their earlobes^2^.

There are a number of causes and/or alterations that require surgical treatment. Among them, we can mention^3^, ^4^:

• Ear lobe clefts or lacerations secondary to trauma;
• Congenital alterations;
• Facial aging;
• Keloids;
• Ear lobe tumors.

Many techniques have been described for the correction of ear lobe clefts^1^, ^5^, ^6^, ^7^:

• Direct suturing;
• Zetaplasty;
• Ritidoplasty with ear lobe correction;
• V-shaped flaps;
• L-shaped flaps;
• Other techniques that may very well be the ones mentioned above but with a few variations or even a combination among them.

The goal of the present study is to present and discuss the technique used in the Department of Otorhinolaryngology of the School of Medicine of the Federal University of de Uberlândia (FAMED-UFU) and of the Santa Genoveva Hospital, in the treatment of ear lobe clefts.

## MATERIALS AND METHODS

### Patients

We assessed 25 patients from January of 2003 to May of 2005 who had ear lobe clefts, adding up to a total of 35 ears. All patients were females, with ages varying between 16 and 58 years (average of 24.5 years). As to skin color, 10 were white, 12 were brown and 3 were black.

Patients were then sorted in two groups:

• Full cleft: usually unilateral, caused by direct local trauma, for example: sudden earring pulling, causing total transection;
• Incomplete cleft: usually bilateral and more frequent in the elderly women who have worn heavy earrings for many years. These patients were further divided in 3 groups:
  1. Type I: the cleft extension does not go beyond half the distance between the initial orifice and the lower border of the ear lobe;
  2. Type II: the cleft extension goes beyond half of the distance between the initial orifice and the lower ear lobe border;
  3. Type III: Progressive cleft extension until it becomes a complete defect.

The present study has been approved by the Ethics Committee under protocol # 015/05.

### Surgical Technical

In all 35 cases we used the surgical approach described below, which we call “Earring Approach”.

**Figure 1:**
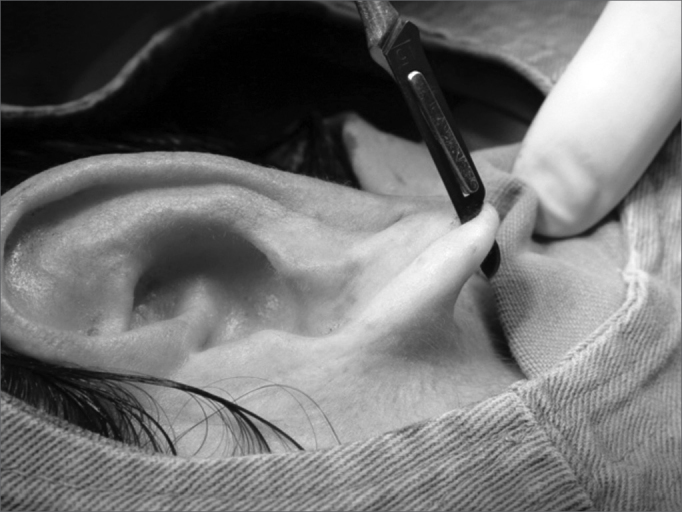
Photograph showing a longitudinal incision completing the earlobe cleft towards the lower border, splitting the lobe in 2 halves.

1. Area cleaning and disinfection with topic povidone;
2. Local anesthesia with 2% lidocaine without vasoconstrictor;
3. Longitudinal incision completing the earlobe cleft towards the lower border, splitting the lobe into two halves (Figures 1 and 2);

**Figure 2:**
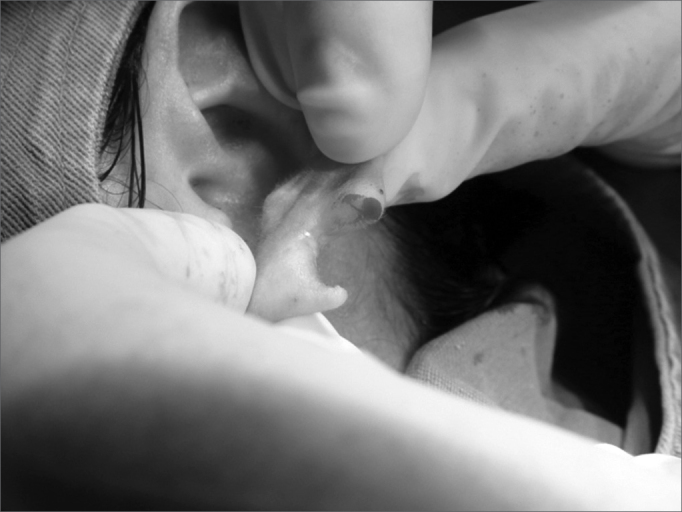
Photograph showing the earlobe split in two flaps.
4. Scaring one of the lobe flaps, thus creating an open wound (Figures 3 and 4);

**Figure 3:**
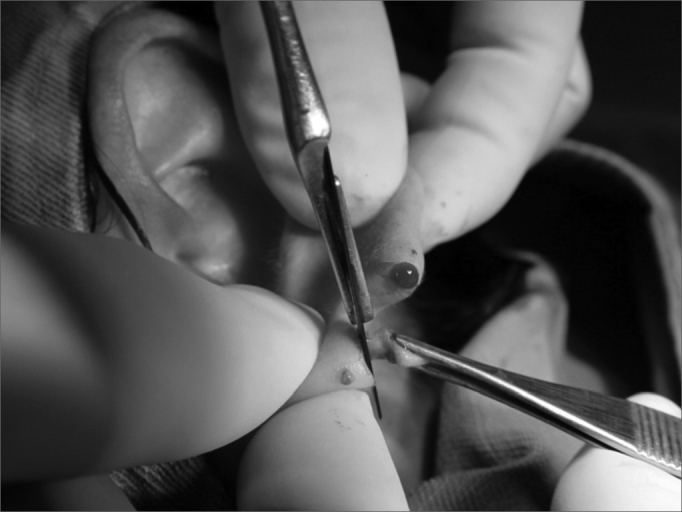
Photograph showing scraping o the anterior lobe flap.

**Figure 4:**
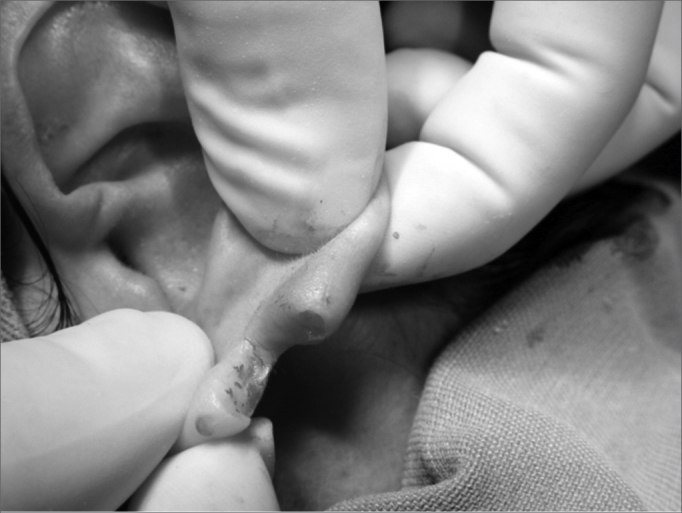
Photograph showing anterior lobe flap with an open wound in the posterior face.
5. Longitudinal incision on the other lobe flap, with a 2.0mm thickness and creating a 5.0 × 2.0mm flap (keeping one of the faces epithelized) (Figure 5);

**Figure 5:**
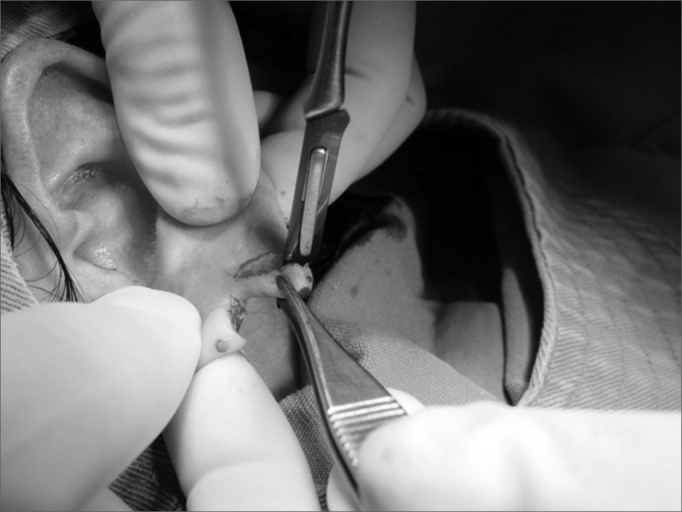
Photograph showing a longitudinal incision on the posterior lobe flap, of 2.0mm thickness, creating a new 5.0×2.0 flap.
6. 2.0mm cross incision on the lower flap border (Figure 6);

**Figure 6:**
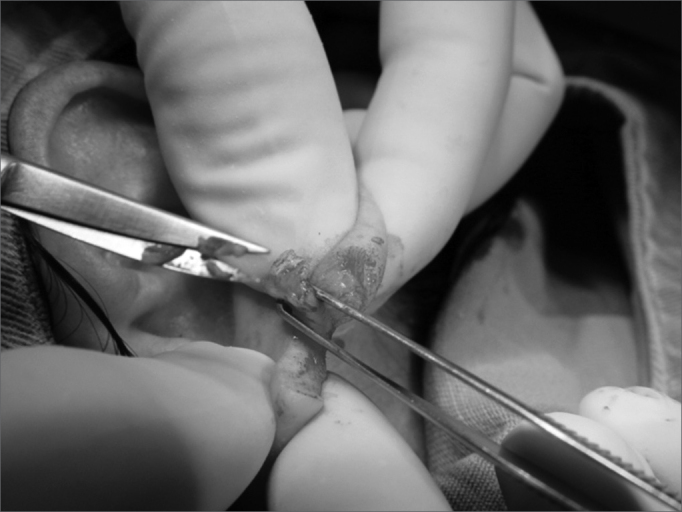
Photograph showing a cross 2.00 incision on the lower border of the central flap.
7. Flap rotation in such a way that the epithelized borders make up the lobe orifice and mononylon 5-0 suturing (Figure 7);

**Figure 7:**
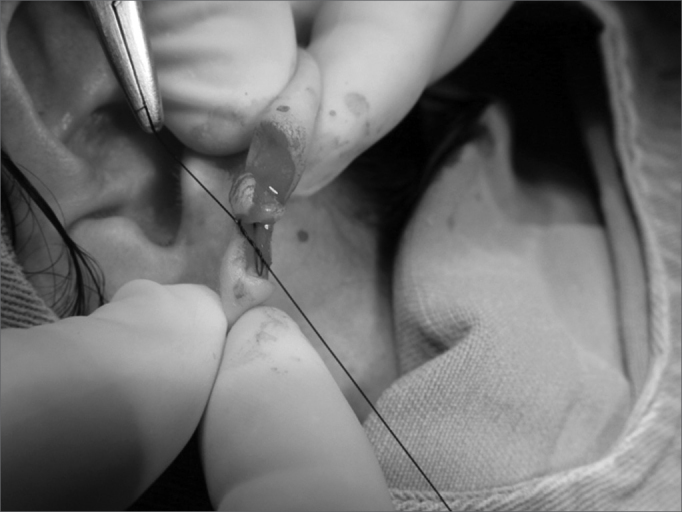
Photograph showing a central flap rotation in such a way as that the epithelized borders make the lobe orifice and mononylon 5-0 suturing.
8. Lobe flaps suturing, starting by the lower free margin with mononylon 5-0 (Figures 8, 9 and 10);

**Figure 8:**
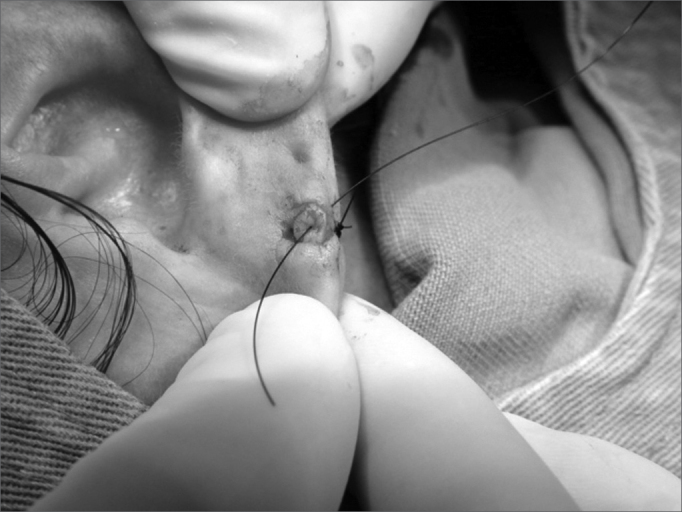
Photograph showing lobe flap suturing through the lower free border with mononylon 5-0.

**Figure 9:**
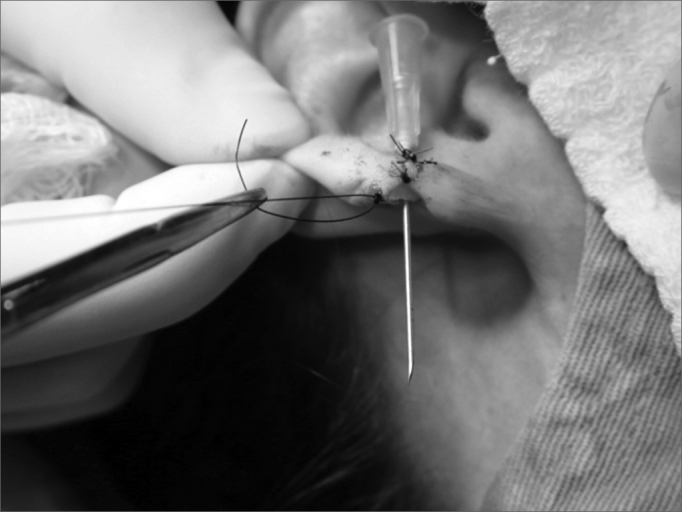
Photograph showing lobe flap suturing with 5-0 nylon and the needle in the final orifice for an earring placement.

**Figure 10:**
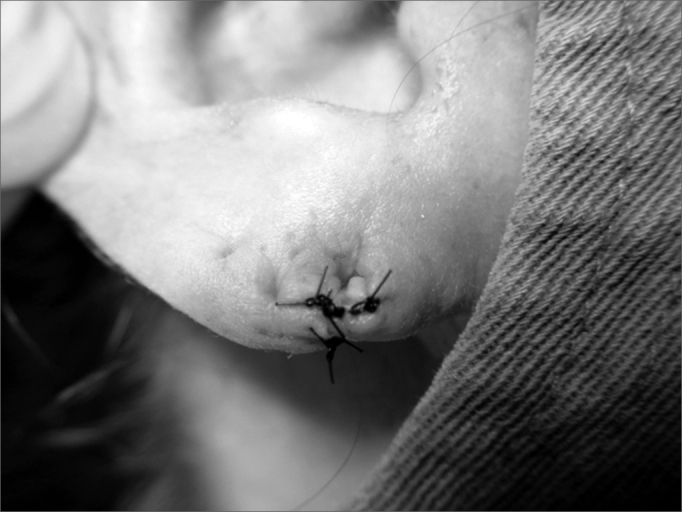
Photograph showing the final surgery outcome. We see the orifice for earring placement, totally epithelized.
9. Micropore dressing;
10. Patients were discharged and prescribed cephalexin and dipirone. They returned after 10 to 15 days for stitch removal and after 30 and 60 days of postoperative for assessment. On the last visit the surgery was assessed by both the physician and the patient.

## RESULTS

Of the 35 ears submitted to the “Earring Procedure”, 22 had a complete cleft and 13 incomplete clefts, and of these, 3 had type I clefts and 10 had type II clefts.

**Chart 1:**
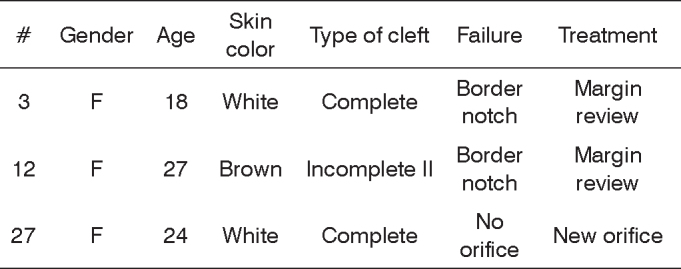
Distribution of the patients who underwent ear lobe surgical treatment and presented with functional/cosmetic impairment.

The final subjective evaluation of the procedure depends on the views of the patient and the surgeon, and in some cases they disagree. Of the 35 cases, 32 presented satisfactory results, 1 had a cosmetic deficit of which only the surgeon noticed and 2 had cosmetic deficits noticed by both the surgeon and the patients, requiring a “second look” procedure (Chart 1).

A second procedure was necessary in these two cases in which there was a level difference in the free margin. Such complication must be avoided with simple suturing techniques, making the wire enter closer to the border of the higher side and farther from the border on the lower side. The other deficit case was a functional one, due to the closure of the lobe orifice; however, the patient did not notice it. If this is the case, a new orifice may be punched in the traditional way, 30 days after surgery.

There were no complications such as keloids, hypo or hyperpigmentation, incisional granuloma, suture dehiscence and skin necrosis.

## DISCUSSION

Many are the techniques that have been described for the surgical treatment of ear lobes. In 1954, McLaren suggested a mild scraping of the cleft borders and simple margin suturing^2^, ^5^. Besides not keeping the lobe orifice, simple suturing favors the formation of a notch.

In 1961, Boo-chai, proposed the excision of part of the cleft borders and suturing below the original orifice. In 1973, Pardue proposed the resection of the cleft borders, leaving a piece of skin on the upper part of one of the sides, which will be used to build the lobe orifice^3^, ^5^. Although attempting to keep the orifice, these two techniques favor the notch formation or a level difference in the lower lobe margin.

In 1975, Hamilton and La Rossa described a similar technique to Pardue's, associated to a zetaplasty in the attempt to minimize the notch formation. Although this new approach tried to keep the orifice open and avoid the notch, it is technically more challenging than the approach described in the present paper. In 1978, Argamasso, described a similar approach which left intact skin near the original orifice, and in each half of the lobe it created two triangular flaps, to be sutured afterwards^3^, ^5^.

In 1982, Harak, proposed tissue excision on the anterior face of one of the borders. And then, the same amount of tissue is removed on the posterior face of the other border. This approach also did not preserve the lobe orifice^5^.

Kalimuthu et al. proposed the “V”-shaped flap approach. Fatah (1985) e Fearon & Cuadros (1990), proposed the “L”-shaped flap, which also does not keep the lobe orifice open^2^. We may then see that none of the existing techniques manages, at the same time, to provide satisfactory results like the ones obtained through the “Earring Approach”. This technique allows for a good cosmetic result, without notches or free margin unleveling, while maintaining a strong lobe orifice, and moreover it is technically easier. Unsatisfactory results were considered to be within expectations and of low complexity, in other words, they were easily corrected and the patients were pleased with the final result. There was no relation between failure and skin type, gender or cleft type.

## CONCLUSION

We believe the “Earring Approach” to be an innovative technique, easily performed and bearing good cosmetic and functional results, thus being, as the authors see it, the technique of choice for ear lobe cleft correction.
